# SICTIN: Rapid footprinting of massively parallel sequencing data

**DOI:** 10.1186/1756-0381-3-4

**Published:** 2010-08-13

**Authors:** Stefan Enroth, Robin Andersson, Claes Wadelius, Jan Komorowski

**Affiliations:** 1 Department of Cell and Molecular Biology, The Linnaeus Centre for Bioinformatics, Uppsala University, Box 598, SE-75124 Uppsala, Sweden; 2 Department of Genetics and Pathology, Rudbeck Laboratory, Uppsala University, SE-75185 Uppsala, Sweden; 3 Interdisciplinary Centre for Mathematical and Computational Modelling, Warsaw University, PL-02-106 Warszawa, Poland

## Abstract

**Background:**

Massively parallel sequencing allows for genome-wide hypothesis-free investigation of for instance transcription factor binding sites or histone modifications. Although nucleotide resolution detailed information can easily be generated, biological insight often requires a more general view of patterns (footprints) over distinct genomic features such as transcription start sites, exons or repetitive regions. The construction of these footprints is however a time consuming task.

**Methods:**

The presented software generates a binary representation of the signals enabling fast and scalable lookup. This representation allows for footprint generation in mere minutes on a desktop computer. Several different input formats are accepted, e.g. the SAM format, bed-files and the UCSC wiggle track.

**Conclusions:**

Hypothesis-free investigation of genome wide interactions allows for biological data mining at a scale never before seen. Until recently, the main focus of analysis of sequencing data has been targeted on signal patterns around transcriptional start sites which are in manageable numbers. Today, focus is shifting to a wider perspective and numerous genomic features are being studied. To this end, we provide a system allowing for fast querying in the order of hundreds of thousands of features.

## Background

Massively parallel sequencing is rapidly becoming the gold standard in hypothesis-free genome-wide studies of for instance transcription factor binding sites [1,2] histone modifications [3-5] and nucleosome positioning [6,7]. In such large-scale studies, the general pattern of these events are often sought/monitored around distinct genomic coordinates such as transcription start/end sites or other biological features such as transcription factor binding sites. The creation of gene-centred footprints can be a computationally intensive and time consuming process, e.g., the most recent human ensembl database [8] lists 23'438 genes corresponding to 140'426 annotated transcripts and 528'281 exons. Furthermore, as standard off-the-shelf desktop computers often come with hundreds of gigabyte of hard drive storage space is not an issue. With this in mind, we designed a simple program suite that stores the fragment count (overlaps) in a binary format suitable for fast access and recovery. To the best of our knowledge, no other program suites for sequence data are tailored against the task of producing footprints. There are several other applications (e.g. [9-11]) for visualizing complementary components such as individual fragments including alignment mismatches against reference sequences, or, that allows for interactive browsing through the data, which SICTIN does not. Since we only store the pileup information and the program suite is designed with access/lookup speed as the primary goal we chose a binary representation of every base pair along the genome. In addition, although this representation introduces overhead disc usage where there are no aligned fragments, this solution makes accessing and footprint calculation fast and easy.

### Implementation

The program suite consists of two major parts, i) the program that creates the binaries, *build_binaries*, where the fragment overlap counts (pileups) are stored and ii) programs that access the signals at given coordinates. The latter category has two sub-programs each designed to perform slightly different tasks, one which pulls regions specified by start and stop coordinates (*access_signal*) and one which computes footprints (*make_footprint*), i.e. average signals over given regions centred on specific locations with respect to transcriptional direction (strand).

### Building the binaries

The *build_binaries* program currently accepts four input formats. Firstly, files in the SAM [12] format which is becoming the standard format for representing aligned fragments. Secondly, a text file (GFF) with columns specifying sequence, start, stop, orientation and a possible count/score of the read. The user can specify which data is given in each column and what character (or string) that separates the columns in the file. These input files need not be ordered in any way, although the building times are much shorter using sorted input since ordering reduces the time used to position the physical heads on the hard drive by the underlying file system. The program also accepts BED and WIG formatted tracks from the UCSC Genome Browser [13]. In case of BED files, which are 0-based left-closed and right-open, the resulting binary files will be 1-based and closed.

The program creates separate binary files for each reference sequence listed in the input file and for fragments aligned to the sense (forward) and anti-sense (reverse) strand of the reference sequence. If a combined signal - where the fragments are prolonged to a biologically relevant length, e.g. size of sonicated fragments - is desired, an additional file containing the combined signal is created with a prolongation length defined by the user. The user can also choose to store only the start coordinates of the fragments. Finally, a text file containing some basic statistics (high/low coordinates, number of fragments) of the run is also produced. All user changeable parameters are described in Additional file 1, Table S1.

### Accessing the binaries

In each created binary file we first store an offset indicating the first (lowest) genomic coordinate of the input fragments and then find a given coordinate by moving a file pointer in the binary file according to this offset. This gives almost constant access to any coordinate in a given sequence i.e. chromosome. We provide two main access modes; looking up specific regions with given start and stop coordinates or providing averaged signals over several coordinates with respect to strand information, i.e. footprints. In the footprint-case, the resulting signals are always given in the transcription-direction of the queried gene/location. The user can also choose to report only regions with counts above a certain threshold or moving all queries by a specified distance. The latter could be used as negative control e.g. to detected transcription factor binding sites.

## Results

### Build and accessing times

**Figure 1:**
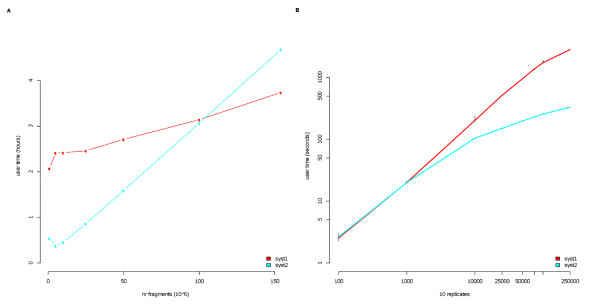
**Build and Access times**. **A **Time in hours spent building the binaries using 1 M to 150 M fragments. 'syst1' was a desktop pc (Dell Precision 390) with Windows Vista using a single CPU running at 2.4 GHz, 'syst2' was a single computational node (Xserve) with Mac OS X Server running at 2 GHz. **B **Time spent in seconds executing footprint queries using 100 to 250'000 unique locations spread uniformly across chromosomes. Each point correspond to the average over 10 different replicates, the black line indicate maximum and minimum value for the replicates.

We tested the programs using large public human datasets [6] with hundreds of millions of sequence fragments. The build time spent on two different test systems are shown in Figure 1A. We find that these large data sets can be processed within hours. The main goal of this work was to present fast ways of retrieving data and the signals are thus represented at every genomic position by a single number. Each binary file contain a coordinate offset and thus any desired location in chromosome is rapidly accessed by moving the file pointer across the binary file with respect to this coordinate offset. The number of overlapping fragments that can be represented at each position depends on the size (number of bytes) that the particular platform/compiler reserves for each position. Per default, SICTIN uses a data type (unsigned short) that is represented by two bytes allowing up to 65536 (2^16) overlapping fragments per position. If any position should supersede this number the value of that position is truncated at 65536. The total number of truncated positions is reported in the basic statistics file produced. The type can be changed in the make-file before compilation if larger - or floating point - numbers are desired. The floating point format allows using this system for microarray data, although this would mean preparing the input so that overlapping probes/tiles are not counted multiple times. For instance, using one unsigned short per position in the human genome requires 250 million shorts for chromosome one, or roughly 500 million bytes, which is around 0.5 GB. Since we use separate files for forward, reverse and combined signals, the total disc space used for this chromosome is around 1.5 GB. In this example the whole human genome required 16.7 GB of disc space. Note that this figure is not directly related to the number of sequenced fragments or length of these, but rather the genomic interval that the fragments were aligned in. A rough estimate gives that the used storage space would be equal to input-files storage space at around 80 million reads for the SAM-format and 400-500 million reads for a minimum GFF/BED-format depending on the amount of information stored.

To test the access times, we generated sets of randomly (uniform) distributed queries over the whole genome with a uniform distribution over sequences (chromosomes) in order to enforce many shifts in signal sources. In Figure 1B the average lookup times over 10 replicates using 100 to 250'000 queries are depicted. Even complex queries with 10 replicates - for instance expression classes - of thousands of features can be completed in 15 minutes.

### Case study A

In a recent study [14] we examined the binding patterns for 38 previously published human data sets of nucleosome location and various histone modifications such as methylations and acetylations. Previously, these modifications had been mapped out on and around transcription start sites (TSS). We decided to take this analysis one step further and investigated all binding patterns on and around all exons thereby scaling the number of genomic features by a factor 10. We were also able to rapidly create footprints split both on the length of the exon and on the expression of the exon/gene. In total we produced on average 6 different footprints for each of the 38 factors investigated each based on tens of thousands of genomic locations. Once the binary representation of the signals was in place we could also easily explore other relevant biological questions. In the following two sections the nucleosome data of human resting T-cells from Schones *et al. *[6] was used.

#### Micrococcal nuclease specificity

**Figure 2:**
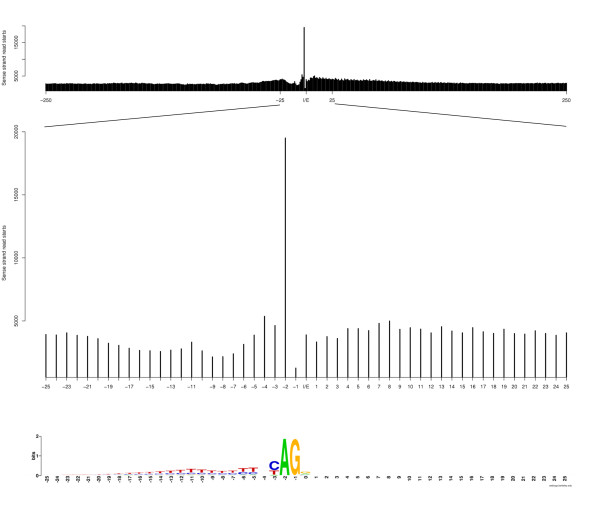
**MNase cut-preferences**. Number of sense-strand fragments starts at intron-exon junctions +/- 250 nucleotides (top) and a zoom of +/- 25 nucleotides (middle). The cut-pattern of MNase is co-located with the conserved donor site in the last 2 nucleotides in the intron (bottom).

The enzyme, Micrococcal nuclease (MNase), often used to digest non-nucleosome DNA, has a pronounced sequence specificity [15]. A cut is much more likely to occur at the 5' end of an A (or T) than G (or C). We created footprints of the nucleosome data around intron/exon junctions using only the fragment starts of the reads ('-so' option to *build_binaries*). From Figure 2, it can be seen that a much higher fraction of reads start at the 5' end of the highly conserved 'A' in the AG-donor site just upstream of the exon. The nucleosome signal peak centred over exons cannot however be explained by this bias. We artificially scaled down the number of reads with a starting point at -2 compared to the exon to the mean over the +/- 250 bp region (without the -2 count) and re-generated the intron-exon junction footprint (Additional file 1, Figure S1). Although slightly less prominent, there is still a peak situated at the same location. Recently, Tolstorukov *et al *[16], also addressed this MNase-bias when analysing positions of H2A.Z and histone 3 lysine 4 trimetylated nucleosomes and found that this bias did not, in principle, affect their results.

#### TSS revisited

**Figure 3:**
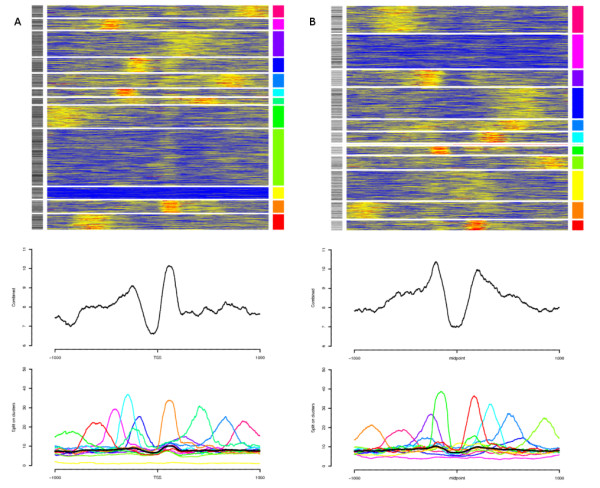
**Nucleosome positioning around TSS (revisited)**. **A **Nucleosome binding patterns around the top 1.5 k expressed TSSs by exon-expression. No alternative TSSs were allowed in the plotted regions. In the top panel, the individual signals (+/- 1 kb) are clustered. The grey scale indicator to the left correspond to length of first exons, light to dark (shorter to longer). The mid-panel shows the resulting footprint for all signals and the bottom panel footprints for the individual clusters. Colors correspond to the rightmost color guide in the top panel. The black line is the combined signal. **B **Nucleosome binding patterns centred between bidirectional genes found at most 1 kb apart. Panels as in A. Clusters with less than 5 members (1 found) are excluded from the bottom-panel. The grey scale indicator to the left correspond to distance between bidirectional TSS's, light to dark (closer to distant).

The nucleosome pattern over TSS's of expressed genes has previously been described as to be well positioned and ordered, i.e. phased, with respect to the TSS's [6,17]. To investigate this on an individual TSS level we collected nucleosome data from +/- 1 kb of the top 1'500 expressed first exons using exon expression array data [18]. The individual signals were then clustered with k-means using 12 clusters. We chose 12 clusters as a 2000 bp window would fit 12 nucleosomes and their linkers. We found that the phasing seen in a combined footprint does not generally reflect the nucleosome positioning on the individual level (Figure 3A). Instead, the majority of the individual regions seem to contain only one or two strongly positioned nucleosomes anywhere in the window. Surprisingly, the +1 nucleosome, which has the strongest signal in the combined footprint, does not have a strong presence in clusters where there is another highly indicated position. The +1 nucleosome does, however, have some signal in all clusters with any present signal. Histone modifications around bidirectional promoters have previously been investigated, Rada-Iglesias *et al *[19] found a bimodal pattern of H3ac around such promoters and Lin *et al *[20] proposed that bidirectional promoters should be nucleosome-free based on the signals of several histone modifications. We extracted all Ensembl genes having a bidirectional conformation, which was defined here as gene starts on different strands separated by at most 1 kb. We then extracted the signals centred on the midpoint of each such bidirectional pair. The resulting footprints are shown in Figure 3B. In concordance with previous results, the nucleosome signal displays a clear bimodal pattern around a nucleosome depleted region. Furthermore, the same pattern as around high-expressed genes (Figure 3A) with single, rather than several phased, nucleosomes is present. Based on these observations and the fact that as many as 50% of the human promoters might be in a bidirectional conformation, including mRNAs and spliced ESTs in the antisense direction [19] it is likely that much of the observed nucleosome signal upstream of the TSSs is due to genes in a bidirectional conformation. The fact that these upstream signals have less intensity in a combined footprint is due to the synchronization of the signals to only one of the TSSs in the bidirectional pair.

### Case study B

The so called mappability, or uniqueness of the genomic sequence itself is an important factor in the alignment process for next generation sequence data. Less unique regions in a genomic sense are likely to receive lower counts since reads that align to too many genomic locations are excluded. We decided to compare the so called mappability to a RNA-seq data set. The mappability used here was constructed from a wig-file representation of the *Broad alignability *track exported from the UCSC genome browser [13]. This data was generated as part of the ENCODE project [21]. The RNA-seq data was downloaded from the example data collection available from the SOLiD Software Development Community website [22]. The RNA-seq data consisted of around 175 M aligned fragments.

**Figure 4:**
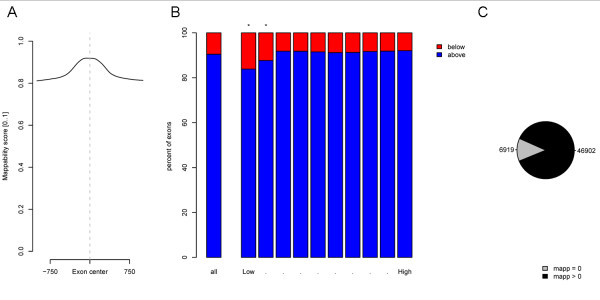
**Effects on mappability on RNA-seq data**. **A **Footprints of mappability scores over all human exons. **B **Proportion of exons that have an average mappability score above or under the genome average mappability score both for all exons and in 10 groups of exons split on RNA-seq signal (Low..High). Each expression group contain the same number of exons. Groups with significantly (p < 0.001, based on the binomial distribution) higher proportion of low mappability scores than the genome average are marked with an asterisk (*). **C **In the groups selected as significant in (B), the number of exons with an average of zero (0) mappability scores and non-zero mappability scores are shown.

First we produced exon centred footprints of the mappability score (Figure 4A), and it is clear that the exonic sequences have, on average, a higher mappability than the surrounding intronic regions. The average mappability score (ranging from 0 to 1) over all human exons is 0.92. We then continued by calculating the average RNA-enrichment and mappability score individually for each of the 290 k+ human exons listed in ensembl[8]. This was done by using the *access_signal *program with the "-avg" option with a single query file per chromosome. The resulting values where grouped in 10 bins according to the RNA-seq signal. For each of these bins the corresponding mappability scores were collected and the fraction of individual exons that had mappability above or below the genome average was recorded. The results are plotted in Figure 4B and we found a significant over-representation of low mappability scores in the groups with low RNA-seq signal. For the two significant groups, we continued by looking at the number of exons with a zero-mappability score and found around 7 k exons (Figure 4C). For these exons the low RNA-seq enrichment is not necessarily due to low expression but can also be an effect of the underlying sequence composition. This illustrates the need to take mappability into account when analysing next generation sequencing data.

## Conclusions

The advent of massively parallel sequencing technologies has opened a field for hypothesis-free investigation of e.g. protein-DNA interaction. In order to facilitate truly exploratory biological data mining we have designed and implemented a system where footprints over genomic features ranging in the order of hundreds of thousands can be rapidly constructed once the binary representation of the signals has been built. Such investigations could include signal over microsatellite repeats, ultra conserved regions or exons. Since the program suite also parses for instance the WIG-format, the analysis can easily be extended to footprinting GC-content or analysing any annotation track from the UCSC repositories [13].

## Availability and requirements

• **Project name: **SICTIN, source code available in 'Additional file 2'

• **Operating system(s): **Platform independent, tested on Windows XP/Vista, Mac OS X Leopard, Linux (CentOS 5)

• **Programming language: **C/C++

• **Licence: **Lesser GNU General Public License (LGPL)

## Competing interests

The authors declare that they have no competing interests.

## Authors' contributions

SE designed and implemented the programs and wrote the manuscript. RA participated in the design of the programs and in the case-studies. CW and JK coordinated the studies and were involved in writing the manuscript. All authors read and approved the final manuscript.

## Supplementary Material

Additional file 1 **Supplementary material**. The supplementary material contains one additional figure, a complete description of user parameters to the programs, compile instructions of the programs and usage examples. Click here for file

Additional file 2 **Source code to the programs**. Complete source code and make file to the programs in a single tar-archive. Click here for file
