# Supplementary material for: SICTIN: Rapid footprinting of massively parallel sequencing data
Source: BioData Min. 2010 Aug 13;3:4. doi: 10.1186/1756-0381-3-4 (PMC2928217; doi:10.1186/1756-0381-3-4)
Supplement: Additional file 1 — Supplementary material. The supplementary material contains one additional figure, a complete description of user parameters to the programs, compile instructions of the programs and usage examples. [file 1756-0381-3-4-S1.DOC]

# Supplementary.

### Compile instructions.

For systems with make installed, simply change to the SICTIN directory and type ‘make’. To compile manually, follow the example in the README-file

### Usage examples.

Starting from a .BED file called ‘input.BED’, building the binaries with a combined overlap signal with 180 bp use the following options:

<local path>/SICTIN/bin/build_binaries –BED –i input.BED –p <path to where the binary files should be stored, needs to exist> -bl 180

If the input is a comma separated GFF-file with sequence name in column 1, strand in column 2, start & end in column 3 & 4 the call would be:

<local path>/SICTIN/bin/build_binaries –GFF –i input.gff –p <path to where the binary files should be stored, needs to exist> -bl 180 –d -si 1 –fs 3 –fe 4 –sc 2

Accessing a single location [1000,1500] in binary file called ‘F_chr1.bin’, storing the output in a file called ‘tmp.txt’ would be:

<local path>/SICTIN/bin/access_signal -i F_chr1.bin -from 1000 -to 1500 -o tmp.txt

The contents of ‘tmp.txt’ will be:

> 1000-1500

0 1 1 2 2 2 4 …

If multiple locations was queried using the ‘-q’ option, the resulting output will have one such row per location. Failed queries, e.g. outside of the input range, will be written to the error file (default ‘err.txt”).

<local path>/SICTIN/bin/access_signal -i F_chr1.bin –q temp.q -o tmp.txt

‘temp.q’

1000 1500

6000 7000

1200 1700

‘tmp.txt’

> 1000-1500

0 1 1 2 2 2 4 …

> 1200-1700

1 2 3 4 5 2 1 …

‘err.txt’

6000 7000

If the ‘errSymb’ is specified, the headers (“> 1000-1500”) will be excluded and rows of failed queries will instead only contain this symbol. e.g

‘tmp.txt’

0 1 2 2 2 2 4 …

NA

1 2 3 4 5 2 1 …

To create a footprint the queries only contain sequence names, one center position and strand information per row, the size of the window around this position is determined by the ‘-d’ option. The call and result would be:

<local path>/SICTIN/bin/make_footprint -ip <path to where the .bins are> -q temp2.q –d 500 –o mysigs.txt

The produced output files will be called ‘F_mysigs.txt’ and ‘R_mysigs.txt’ and contain the signals build from fragments match to the sense (F) and antisense (R) strand respectively. If the combined signals was build these are access using the ‘-c’ options and would be called ‘C_mysigs.txt’.

temp2.q:

chr1 145 +

chr2 500 -

chr1 454 +

F_mysigs.txt:

> + 2

0.5 0.5 1 1 1.5 0 …

>- 1

0 0 0 1 1 1 1 1 …

Each of the output files contains 4 rows. The first 2 rows contain data for features on the sense (+) strand, and the last two for features on the anti-sense strand (-). **Note that the signals for the features on the anti-sense strand have been reversed so that the first number is upstream of the TSS in the direction of transcription.** In this case 2 queries were successfully executed on the sense strand and 1 on the anti-sense strand. Failed queries are written in the error file as described above.

**Table S1. Summary of the programs and input parameters.**

| **build_binaries** | | |
| --- | --- | --- |
|  | **-i** | infile, containing the sequenced reads, required. |
|  | **-o** | outfile stub, prefixes ‘R’,’F’ (and ‘C’) will be added, optional. |
|  | **-bl** | create combined (C) overlap signal, if > 0 with this length. def 0 |
|  | **-p** | outfile path, where files are to be placed, optional. |
|  | **-so** | store only start position of the fragment, default off. |
|  | **-SAM** | infile format |
|  | **-GFF** | infile format, additional parameters to this format below. |
|  | **-d** | delimiter used in the GFF-input file |
|  | **-si** | sequence identifier column in the GFF-input file, default 1 |
|  | **-fs** | fragment start column in the GFF-input file, default 2 |
|  | **-fe** | fragment end column in the GFF-input file, default 3 |
|  | **-sc** | fragment strand column in the GFF-input file, default 4 |
|  | **-vc** | fragment value column in the GFF-input file, if > 0 use this (integer) value instead of 1 as count of the fragment. |
|  | **-BED** | infile format, outfiles will be 1-based, additional parameters to this format below. |
|  | **-si** | sequence identifier column in the GFF-input file, default 1 |
|  | **-fs** | fragment start column in the GFF-input file, default 2 |
|  | **-fe** | fragment end column in the GFF-input file, default 3 |
|  | **-sc** | fragment strand column in the GFF-input file, default 6 |
|  | **-WIG** | infile format, outfiles will be 1-based, additional parameters to this format below. |
|  | **-scale** | scores in the WIG-input file will be multiplied with this value, useful if the scores are real-values and storage format is whole-number. |
| **access_signal** | | |
|  | **-i** | infile, single “.bin” file, required. |
|  | **-o** | outfile, required. |
|  | **-q** | query file, should contain start and stop, tab delimited. |
|  | **-from** | if single query, from this bp |
|  | **-to** | if signle query, to (including) this bp |
|  | **-e** | errorfile, defaults to 'err.txt', contains failed queries |
|  | **-errSymb** | errorsymbol, disables headers in the 'outfile' and 'errorfile' completely. Writes the symbol instead in 'outfile' |
|  | **-avg** | toggle average (mean) signal in each query only (def. no) |
| **make_footprint** | | |
|  | **-ip** | infile path, correspond to ’-p’ for build_binaries |
|  | **-is** | infile stub, correspond to ’-o’ for build_binaries |
|  | **-os** | outfile stub, ‘F_’,’R_’ and (‘C_’) prefixes will be added. |
|  | **-c** | toggle combined (‘C_’), default off. |
|  | **-q** | query file, contains TSS position and strand (-/+), tab delimited, required. |
|  | **-e** | errorfile, defaults to 'err.txt', contains failed queries |
|  | **-d** | offset, how long distance +/- the TSS positions to consider default:5000 |
|  | **-p** | toggle progress meter. default off |
|  | **-s** | score cutoff, at least one bp in the interrogated region must have at least this score to be included. default 0 |
|  | **-m** | move offset, move (downstream) all the queries this many bp, default 0 |


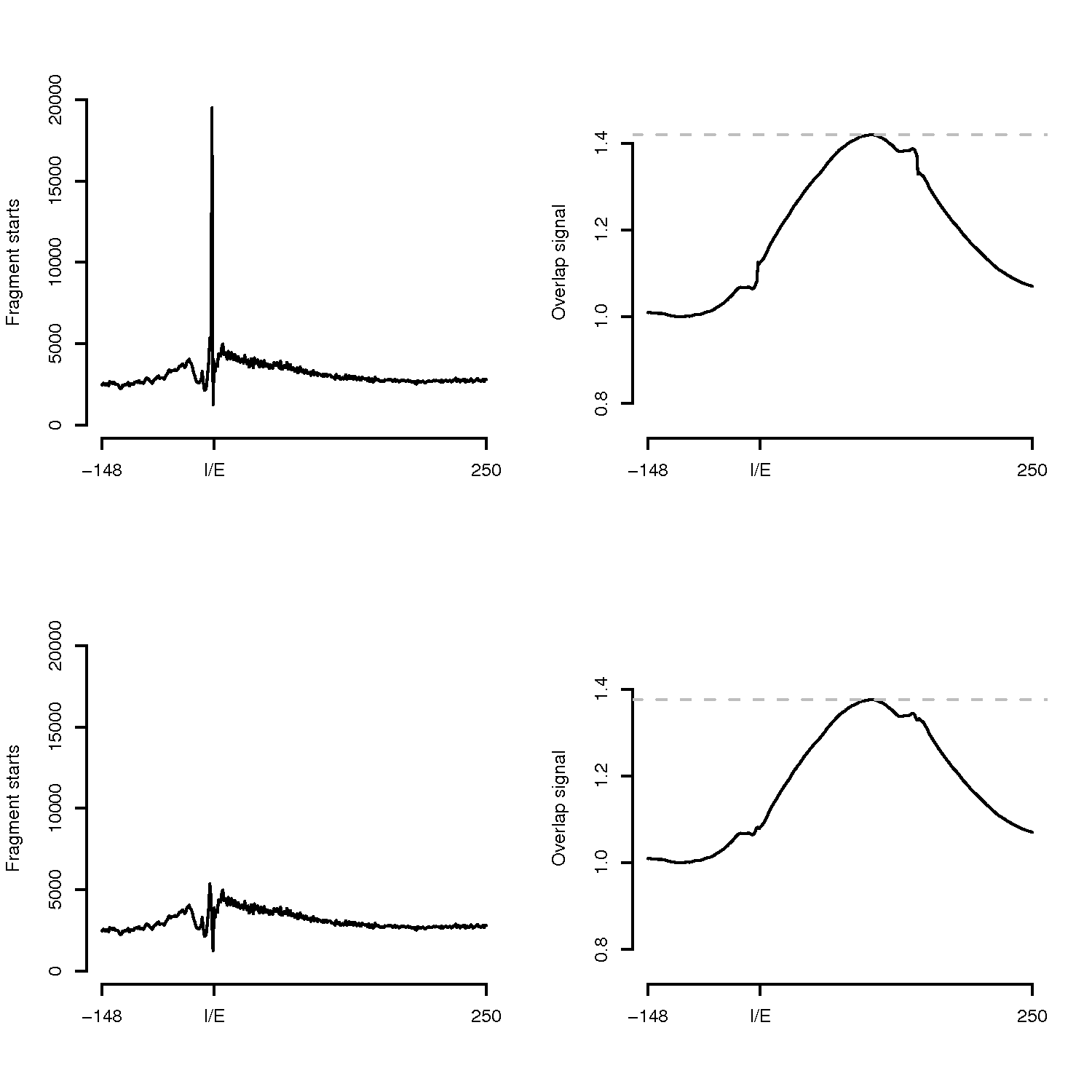


**Figure S1:** **MNase sequence specificity bias on internal exons.**

The high sequence specificity (top row) of the MNase cut on the donor site just upstream of the human internal exons does not account for the nucleosome signal peak over the internal exons (bottom row). The high peak seen in the top left panel was artificially replaced by the mean in the region, excluding the value of the peak (bottom left).
